# Meningioma-associated abscess: an unusual case report and review of the literature

**DOI:** 10.1093/jscr/rjab582

**Published:** 2022-01-17

**Authors:** Sami Rashed, Anna Vassiliou, Rosalie Ogborne, Gráinne McKenna

**Affiliations:** Neurosurgical Department, Royal London Hospital, London, UK; Barts and the London School of Medicine, London, UK; Neurosurgical Department, Royal London Hospital, London, UK; Neurosurgical Department, Royal London Hospital, London, UK

**Keywords:** meningioma, neurosurgery, tumour abscess

## Abstract

Central nervous system (CNS) infection and neoplasm occur most often independently. Their concomitant presentation has been noted across different CNS tumours but is considered a rare entity. The phenomenon is mostly seen in relation to direct seeding of infection via frontal air sinuses. Here, we present an unusual case of an occipital meningioma associated with intraparenchymal paratumoural abscess formation. It is also the second documented to culture methicillin-susceptible *Staphylococcus aureus*. We then review and surmise the relevant literature of meningioma-associated abscess. We discuss the clinical presentations, aetiology, suspected pathogenesis, management and outcomes reported.

## INTRODUCTION

Central nervous system (CNS) infection and neoplasm occur most often independently, but their concomitant presentation has also been reported across the literature. A variety of neoplasms have been linked with tumoural abscess formation including glioblastoma, high-grade and low-grade astrocytoma, ependymoma and metastatic lesions. However, a majority of these lesions described intrasellar or parasellar tumours where infection has spread directly from the prenasal cavity, and on the whole, it is considered a rare entity [1]. This case report presents the clinical course and management of a meningioma with related intraparenchymal paratumoral abscesses. We then discuss this in the context of the wider literature as the second case to isolate methicillin sensitive *staphylococcus aureus* (MSSA) within a meningioma.

## CASE REPORT

**Figure 1:**
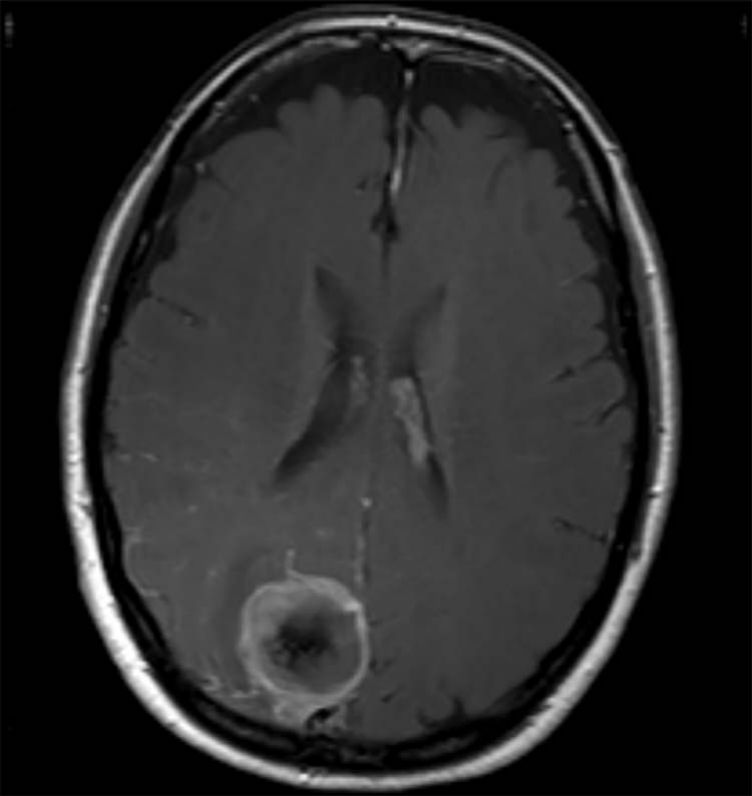
Axial view of a T1 weighted MRI post-gadolinium enhancement demonstrating the appearance of a 3-cm right parieto-occipital extra-axial mass with bony involvement and adjacent small nodule. Most in keeping with a meningioma.

A 52-year-old female presented with headache and visual disturbance. She had no significant past medical history. On examination, a left homonomous inferior quadrantopia was noted. CT and then MRI imaging revealed a solitary 3-cm right parieto-occipital extra-axial lesion with associated dural tail and surrounding oedema (Fig. 1). Her headaches improved with a short course of steroids and the neuro-oncology MDT recommendation was for surgical excision of the suspected meningioma.

**Figure 2:**
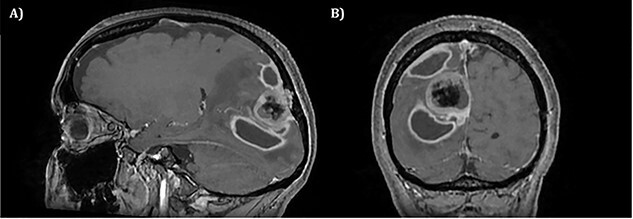
T1 weighted MRI postgadolinium enhancement in both sagittal (**A**) and coronal (**B**) views. There are two new rim-enhancing cystic lesions. A superior lesion of 31.4 mm as measured on the coronal reconstructed images and is abutting the adjacent dura of the right parietal lobe and is sited superior and lateral to the right occipital lesion. Another lesion sited inferior to the known right occipital lesion is the larger of the two lesions and has a septation within it. This measures approximately 34.4 mm on the coronal reconstructed images. On the sagittal images, it can be seen that the anterosuperior enhancing border of this lesion has a thicker and more ill-defined age. There is an adjacent vasogenic oedema.

Two weeks later, whilst awaiting surgery, she attended the Emergency Department with a fever of 38.6°C and mildly raised inflammatory markers (WCC 10.4, Neut 8.4). Urine dip and CXR were unremarkable. The source of the infection was not isolated and the emergency medicine physicians discharged her with a course of empirical oral antibiotics. The neurosurgical team were not made aware of this attendance. Twelve days later, she underwent a planned neuronavigation-planning MRI scan as an outpatient. This was arranged one week prior to the scheduled surgery, in line with our departmental protocol. The MRI was reviewed and two new rim-enhancing collections superior and inferior to the tumour were noted, associated with extensive perilesional oedema (Fig. 2A and B). The patient was then immediately contacted via telephone and reported new worsening of headaches and new right sided weakness. She was admitted to hospital directly for assessment and emergency treatment. Her inflammatory markers on admission had risen to a WCC of 19.5, Neut 16.6, but CRP was <1. She was started on an emergency steroid treatment and subsequently underwent craniotomy, total resection of the meningioma and drainage of the intraparenchymal paratumoural abscesses. Intra-operatively the brain was swollen; pus collections were encountered in abscess cavities superior and inferior to the solid tumour, which had a necroticcore.

**Figure 3:**
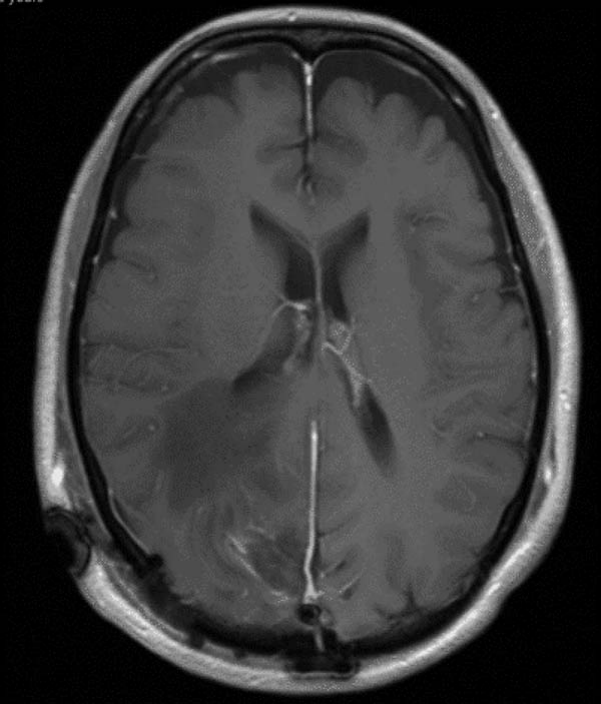
T1 weighted axial MRI postgadolinium enhancement within 48 h post-operatively. Small amount of enhancement within the surgical bed remains. Good resection margins with vasogenic oedema. This may represent a small residual. The rim-enhancing cystic lesions appear to have resolved.

**Figure 4:**
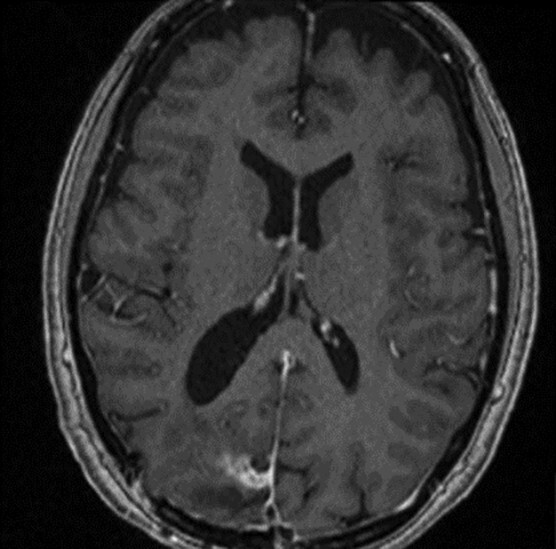
T1 weighted axial MRI post-gadolinium enhancement 8 weeks postdischarge. Right occipital postsurgical appearances are again demonstrated with reduction in the extent of vasogenic oedema. The enhancing rim that was present previously has retracted, with only focal curvilinear enhancement now evident in the right occipital lobe. These appearances are likely postsurgical/treatment related, and there is no convincing residual or recurrent disease.

The operation was successful with a post-operative MRI within 48 h demonstrating complete resection of the tumour and resolution of the cystic lesions, but persisting intraparenchymal oedema (Fig. 3). The patient had an uncomplicated two-day stay in a neurosurgery high dependency unit postoperatively before being stepped down to the general neurosurgery ward. She was reviewed by the microbiology team and worked up for the source of infection. Blood cultures, urine cultures, CXR and transthoracic echocardiography were all negative for a source of infection. Intra-operative pus cultures isolated MSSA. The histopathology of the tumour was Meningioma WHO Grade 1 with large areas of necrosis and secondary abscess formation. She was discharged feeling well 5 days later after a satisfactory biochemical and clinical response. She was given a weaning course of steroids and a 6-week course of intravenous ceftriaxone via a PICC line as an outpatient as per microbiology advice. Ophthalmology assessment 3 months after discharge confirmed a left inferior quadrantonopia and preserved visual acuity. Repeat MRI and clinic review 8 weeks later showed no radiological residual tumour or oedema (Fig. 4), and the patient’s limb function recovered completely but had ongoing visual symptoms.

**Table 1:** Summary of meningioma-associated abscesses reported in the literature included out owncase

Case	Patient	Clinical features	Organism	Meningioma location	Relationship of abscess to meningioma	Histological finding	Grade WHO (2016)	Source of infection	Favourable outcome
Shimomura *et al*. 1994 [2]	64/F	Drowsiness and fever	*Bacteroides oralis*	Right frontal	Intratumoral	Transitional meningioma	1	10 days postgynaecologic surgery	+
Nassar *et al*. 1997 [3]	78/F	Left hemiparesis	*Escherichia coli*	Right occipital	Intratumoral	‘Benign meningioma’	1	Urinary tract infection	+
Eisenberg *et al*. 1998 [4]	78/F	Focal seizure	*Proteus mirabilis*	Left frontal	Intratumoral	Transitional meningioma	1	Urinary tract infection	−
Onopchenko *et al*. 1999 [5]	63/F	N/A	*Staphylococcus aureus*	Left convexity	Peritumoral	N/A	N/A	Recent nephrectomy for abscessed pyelonephritis and drainage of gluteal abscess	+
Yeates *et al*. 2003 [6]	38/F	Seizures, fever, chills and night sweats	*Bacteroides fragilis*	Left frontal	Intratumoral	Meningothelial meningioma	1	3 weeks postvaginal hysterectomy	+
Lind *et al*. 2005 [7]	78/F	Confusion and personality change	*Citrobacter koseri*	Right frontal	Peritumoral	N/A	N/A	Unknown	+
Young *et al*. 2005 [8]	38/M	Headache and fever	Group B streptococcus, *Peptostreptococcus*	Right temporal	Intra and peritumoral	Meningothelial meningioma	1	Dental work	+
Lo *et al*. 2014 [9]	70/F	Left hemiparesis	*E. coli*	Right parietal and left frontal	Intratumoral	Transitional/fibrous meningioma	1	6 days postureteroscopy and lithotripsy	+
Krishnan *et al*. 2014 [10]	55/F	Status epilepticus	*E. coli*	Left frontal convexity	Intratumoral	Psammomatous meningioma	1	Recent urinary stent insertion	+
Moliere *et al*. 2015 [11]	65/F	Headache	*Norcardia novia*	Left occipital	Intratumoral	Meningothelial meningioma	1	Unknown	+
Rao Patibandla *et al*. 2017 [12]	35/M	Headache and vomiting	*Proteus Mirabilis*	Right lateral ventricle	Intratumoral	Transitional type	N/A	Urinary tract infection	+
Sannareddy *et al*. 2018 [13]	56/M	Headache and vomiting	*E. coli*	Left occipital	Intratumoral	Psammomatous meningioma	I	Unknown	+
Sosa-Najera *et al*. 2018 [14]	42/F	Left hemiparesis, focal left seizures and headache	N/A	Right parietal	Intratumoral	Atypical meningioma	II	Unknown	+
Chandra *et al*. 2018 [15]	70/M	Right hemimotor and sensory disturbance	*Streptococcus constellatus*, Fusobacterium, *Prevotella dentalis* and *Parvimonas micra*	Left posterior frontal/parietal lobe	Intra- and peritumoral	Meningothelial meningioma	1	Unknown	+
Ponce-Ayala *et al*. 2020 [16]	63/M	Confusion, aphasia and right hemiparesis	N/A	Left hemispheric	Intratumoral	Anaplastic meningioma	III	N/A	−
Fabbri *et al*. 2020 [17]	76/M	Left sided hearing loss	N/A - ‘sterile’	Right convexity	Intratumoral	Meningothelial	I	N/A	+
Cristopher *et al*. 2020 [18]	75/F	Focal seizures developing to status epilepticus	*E. coli*	Known left frontal and parietal meningiomas	Intratumoral	N/A	I	Urinary tract infection	+
Our Case	52/F	Left Inferior quadrantopia, headache and confusion	*Staphylococcus aureus* (MSSA)	Right occipital	Peritumoral	N/A	I	Unknown	+

WHO = World Health Organisation, M = Male, F = Female, N/A = Not Applicable.

## DISCUSSION

Upon review of the literature with respect to meningiomas associated with tumoral abscess specifically, we identified eighteen cases including our own (Table 1). The most common presenting symptom seen was headache, which was present in six cases including our own [8, 11–14]. Hemiparesis and seizures were also common and seen in four patients [3, 4, 6, 9, 14, 16, 18].

An infective source was identified in a total of ten cases. Six patients had operative interventions, either gynaecological, urological or dental, in the recent period prior to presentation and cultured corresponding organisms [2, 5, 6, 8–10]. The remaining four cases had associated urinary tract infections identified through urine cultures [3, 4, 12, 18]. Despite investigation, eight remaining cases including our own had no clear infective source. One of these cases described a ‘sterile’ abscess formation thought related to androgen treatment in the context of prostate carcinoma [17]. The immunocompromised nature of the patient, recent steroid treatment and presumed urinary/dental infections were cited as potential sources/contributing factors to the remaining cases without a clear cause [7, 11, 13–16].

The organisms cultured were also consistent with a majority abdominopelvic origin. Of the fifteen cases with a confirmed organism, ten were consistent with abdominopelvic microbiota with *E. coli* being the most common organism seen in five cases [3, 9, 13, 18]. The clinical/biochemical inflammatory response seen to infection demonstrated a variable level of severity, from asymptomatic to occult sepsis and also a varied temporal relationship between infection and tumour identification.

As given above, haematogenous spread has been postulated as the most likely pathogenesis of abscess formation in meningiomas. Especially as destruction to the blood brain barrier through open epithelial junctions, gaps between epithelial cells and capillary fenestrations have been demonstrated in meningiomas [9]. The rich vascular supply of tumours, their vascular branching patterns, compression of nearby venous structures resulting in stasis and the nutrient-rich environment have also been cited as potentially contributory [10]. Intratumoral cultures showing very similar sensitivities to those cultured peripherally and our case being one of few describing a lesion remote from the frontal sinuses also strengthens the case [18].

The most common location for abscess formation was within the tumour alone, which was seen in fourteen cases. A peritumoral abscess was seen in two cases and a further two demonstrated the presence of both peritumoral and intratumoral abscesses [5, 7, 8, 15]. One case demonstrated intratumoral abscess formation solely within one of two meningiomas present raising the possibility of preferential seeding of infections depending on vascular supply [18].

The immunocompetent nature and occult source of infection of our case make an interesting point of discussion with the majority of cases occurring with a known infective source, causative procedure or immunocompromised status. As with our case and sixteen of the eighteen cases in the literature, a favourable outcome was achieved. This was defined as complete recovery in nine cases and persistent mild neurological deficit in six cases. All cases required surgical intervention to achieve this and most utilized prolonged antibiotic therapy. Those cases, which mention antibiotic, produce a mean therapeutic duration of 6.7 weeks (range 10 days–12 weeks). Most describe resolution on follow-up imaging with one case requiring repeat surgical intervention in the acute post-operative period due to imaging findings.

In conclusion, we describe the presentation and successful treatment of a rare meningioma and associated intraparenchymal paratumoural abscesses. A phenomenon noted within the literature with evidence suggesting its association to haematogenous spread of classically abdominopelvic organisms. Occult infection, as in our case, is also seen but despite this patients usually have uncomplicated recoveries following surgical intervention and a prolonged antibiotic course.

## CONFLICT OF INTEREST STATEMENT

None declared.
